# Altered γ-Secretase Processing of APP Disrupts Lysosome and Autophagosome Function in Monogenic Alzheimer’s Disease

**DOI:** 10.1016/j.celrep.2018.11.095

**Published:** 2018-12-26

**Authors:** Christy O.Y. Hung, Frederick J. Livesey

**Affiliations:** 1 Gurdon Institute and ARUK Stem Cell Research Centre, University of Cambridge, Cambridge CB2 1QN, UK; 2 UCL Great Ormond Street Institute of Child Health, Guildford Street, London WC1N 1EH, UK

**Keywords:** endosome, lysosome, autophagy, axonal transport, live-cell imaging, Alzheimer’s disease

## Abstract

Abnormalities of the endolysosomal and autophagy systems are found in Alzheimer’s disease, but it is not clear whether defects in these systems are a cause or consequence of degenerative processes in the disease. In human neuronal models of monogenic Alzheimer’s disease, *APP* and *PSEN1* mutations disrupt lysosome function and autophagy, leading to impaired lysosomal proteolysis and defective autophagosome clearance. Processing of APP by γ-secretase is central to the pathogenic changes in the lysosome-autophagy system caused by *PSEN1* and *APP* mutations: reducing production of C-terminal APP by inhibition of BACE1 rescued these phenotypes in both *APP* and *PSEN1* mutant neurons, whereas inhibition of γ-secretase induced lysosomal and autophagic pathology in healthy neurons. Defects in lysosomes and autophagy due to *PSEN1* mutations are rescued by CRISPR-knockout of *APP*. These data demonstrate a key role for proteolysis of the C-terminal of APP by γ-secretase in neuronal dysfunction in monogenic Alzheimer’s disease.

## Highlights

• *APP* and *PSEN1* mutant neurons have deficits in lysosome proteolysis
• BACE1 inhibition rescues lysosome and autophagy defects
• *PSEN1* mutant phenotypes are rescued by genetic deletion of *APP*
• Lysosome and autophagy defects are causes of neuronal dysfunction in AD

## Graphical Abstract

**Figure:**
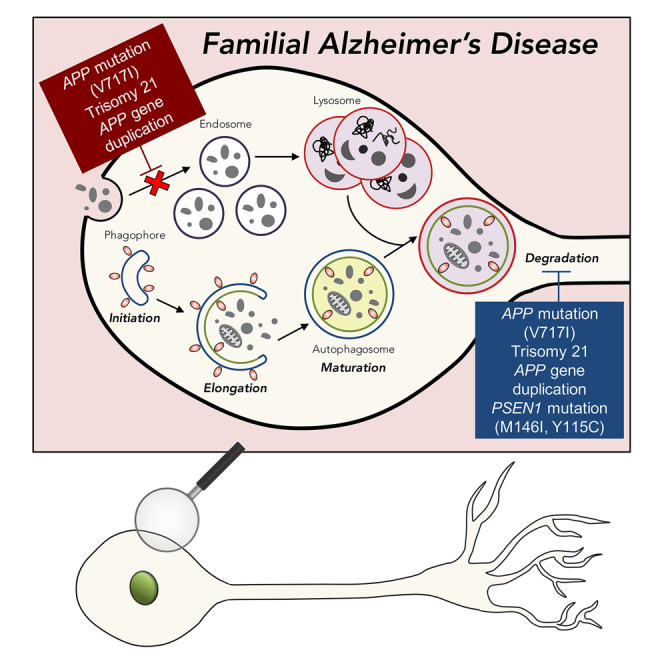

## Teaser

Hung and Livesey demonstrate that mutations in *APP* and *PSEN1* that are causal for early onset Alzheimer’s disease lead to major defects in lysosome function and autophagy in human neurons. The rapid appearance of these defects indicates that they are causes, rather than consequences, of neuronal dysfunction in Alzheimer’s disease.

## Introduction

Abnormalities of the endolysosomal and autophagy systems are both found in Alzheimer’s disease (AD) (Nixon, 2017, Lie and Nixon, 2018). Prominent enlargement of endosomal compartments precedes the appearance of neurofibrillary tangles and neuritic plaques in mouse models of AD (Cataldo et al., 2000). Similarly, selective accumulation of lysosomal dense bodies and autophagic vacuoles in dystrophic neurites has been observed in post-mortem brain samples of people with AD (Nixon et al., 2005), suggesting that autolysosomal proteolysis is impaired in AD (Nixon, 2017). Genome-wide association studies have identified SNPs in several genes with roles in the endolysosomal and autophagy systems as impacting disease risk, including *PICALM*, *CLU*, *SORL1*, *ABCA7*, and *BIN1* (Karch and Goate, 2015). These underscore the significance of the endolysosomal and autophagy systems as a genetic risk hotspot in AD (Nixon, 2017). However, it is not clear whether defects in lysosomal and autophagic systems are a cause or consequence of degenerative processes in AD.

Collectively, genetic data place APP processing by γ-secretase as central to disease initiation in monogenic forms of AD, including familial AD due to *PSEN1* and *APP* mutations (Selkoe, 2002). The majority of known familial AD mutations are autosomal dominant and affect the amyloid precursor protein (*APP*) gene or the catalytic components of the γ-secretase APP-processing complex, presenilin (*PSEN*) 1 and 2 (Bertram and Tanzi, 2008). As well as missense mutations, increased *APP* gene dosage due either to trisomy of chromosome 21 (Ts21) or duplication of the *APP* locus (*APP* (*dup*)) also leads to early onset AD (Rovelet-Lecrux et al., 2006). Conversely, a protective mutation near the BACE-cleavage site in APP has been identified that reduces the risk of developing dementia (Jonsson et al., 2012).

How changes in APP processing by γ-secretase lead to AD is still not well understood. Deposition of amyloid beta (Aβ) peptide fragments of the amyloid precursor protein (APP) in amyloid plaques and hyperphosphorylated tau in neurofibrillary tangles are the cellular hallmarks of AD (O’Brien and Wong, 2011). The amyloid hypothesis proposes that it is the accumulation of extracellular toxic forms of Aβ as both soluble oligomers and insoluble plaques that is the primary pathologic driver of the disease (Hardy and Selkoe, 2002). However, causal links between production of potentially toxic longer forms of Aβ peptides, amyloid deposition, and disease initiation have not been definitively demonstrated and remain contentious (De Strooper and Karran, 2016).

Nascent APP proteins mature through the constitutive secretory pathway from endoplasmic reticulum and the Golgi apparatus and are transported to the plasma membrane (Thinakaran and Koo, 2008). Endosomal and lysosomal compartments are involved in APP processing and perturbation of the endolysosomal system can lead to altered APP processing, Aβ peptide production and amyloid accumulation (Nixon, 2017). Conversely, increased expression of APP or expression of mutant APP in fibroblasts leads to dysfunction of the endolysosomal system (Cataldo et al., 2008). AD mutations in *PSEN1* lead to altered lysosomal function, including reduced acidification through altered PSEN1-mediated recruitment of the Vo ATPase to the lysosome, which in turns leads to defects in neuronal macroautophagy (Lee et al., 2010). Moreover, PSEN1 deficiency has been shown to alter lysosomal calcium storage and release (Coen et al., 2012, Neely Kayala et al., 2012). While the appearance of pathological changes in the endolysosomal and autophagy systems is an important early feature of AD, whether these changes are causes or consequences of primary drivers of neurodegenerative processes remain unclear (Peric and Annaert, 2015).

To address this question, we investigated how AD mutations in *APP* and *PSEN1* affect the endolysosomal and autophagy systems in human-stem-cell-derived cortical neurons. We and others have previously shown that mutations in *APP* and *PSEN1* lead to immediate changes in APP processing, with either increased relative or absolute amounts of longer forms of Aβ peptides (Yagi et al., 2011, Muratore et al., 2014, Moore et al., 2015). We now find that mutations in both *APP* and *PSEN1* causal for monogenic, early onset AD, lead to major defects in lysosome function and autophagy in induced pluripotent stem cell (iPSC)-derived human neurons, with *APP* mutations also disrupting endosomal function. Reducing production of C-terminal APP by inhibition of BACE1 rescued these phenotypes in both *APP* and *PSEN1* mutant neurons, and defects in the lysosomal and autophagic systems due to *PSEN1* mutations are prevented by CRISPR knockout of *APP*. These data demonstrate that compromised proteolysis of the C-terminal of APP by γ-secretase by autosomal dominant mutations in both *PSEN1* and *APP* causes dysfunction of the endolysosomal and autophagy systems in human neurons and is a driver of neurodegeneration in familial AD.

## Results

### AD Mutations in *APP*, but Not *PSEN1*, Result in Early Endosome Abnormalities in Human Cortical Neurons

To study the effects of AD mutations in *APP* and *PSEN1* on the neuronal endolysosomal system, we generated cortical excitatory neurons from iPSCs derived from individuals with genetic changes or mutations causal for AD: *APP* V717I, trisomy chromosome 21 (Ts21), APP duplication (*APPdup*), and three different *PSEN1* mutations (Y115C, M146I, and Intron 4). Neurons were generated according to our previously described methods (Shi et al., 2012a) and confirmed as being cortical in neuronal identity by both gene expression and immunofluorescence (Figures S1A–S1C). As we previously described (Moore et al., 2015), both *PSEN1* and *APP* missense mutations increase the proportion of longer forms of Aβ relative to shorter forms, as reflected in reduced Aβ40:Aβ42 ratios, whereas *APPdup* and Ts21 neurons overproduce all measured forms of Aβ peptides (Figures S1D–S1G).

**Figure 1:**
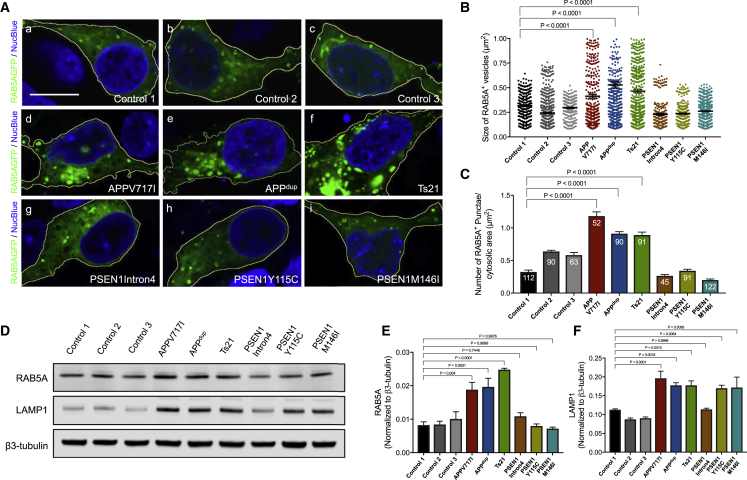
Alzheimer’s Disease Mutations in *APP*, but Not *PSEN1*, Result in Early Endosome Abnormalities in Human Cortical Neurons (A) Representative images of neurons generated from three independent non-demented controls, *APP* (*dup*), *Ts21*, and *APP* V717I and *PSEN1* mutants (Y115C, M146I, or Intron 4) induced pluripotent stem cells (iPSCs). Scale bar, 10 μm. (B and C) Significant increases in the size (B) and number per unit area (C) of Rab5A-GFP positive endosomes in *APP* but not *PSEN1* mutant neurons, compared with non-demented controls (one-way ANOVA with Tukey correction between controls and Alzheimer’s Disease [AD] neurons in each case). Rab5A-GFP^+^ vesicle size measures in (B) were from three independent experiments, including an independent neural induction in each case. Total number of vesicles measured (n): 260 (Control 1), 654 (Control 2), 265 (Control 3), 472 (*APP* V717I), 574 (*APPdup*), 700 (*Ts21*), 250 (*PSEN1* Intron4), 355 (*PSEN1* Y115C), and 620 (*PSEN1* M146I). Number of vesicles per unit cytoplasmic area (C) was measured in three independent experiments (n = 3), with total numbers of cells indicated within each bar. (D–F) Representative western blots of Rab5A, LAMP1, and neuron-specific β3-tubulin in three independent control and AD neurons are shown (D). Levels of Rab5A (E) and LAMP1 (F) were calculated relative to β3-tubulin from three independent experiments (n = 3), including an independent neural induction in each case. Error bars, SEM. See also Figure S1.

Endosomes are highly active processing sites for APP, and accumulation of enlarged early endosomes and lysosomes in neurons has been observed in post-mortem brain samples of patients with sporadic AD and some forms of familial AD (Cataldo et al., 2000, Cataldo et al., 2001, Colacurcio et al., 2018, Nixon, 2017). Using a GFP fusion of the small Rab GTPase Rab5A to visualize early endosomes, we observed significantly enlarged Rab5A-positive early endosomes in neurons with missense mutations in *APP* or increased copy number of *APP (APPdup* and Ts21), compared with those in control neurons (Figures 1A and 1B). Moreover, the number of enlarged early endosomes per neuron was increased significantly in both *APP* duplication and missense mutation neurons (Figure 1C). This was accompanied by upregulation of the total mass of early endosomes, as reflected in an increase in endogenous Rab5A protein levels (Figures 1D and 1E).

In contrast, endosomal number and size, as measured by imaging and western blot levels of Rab5, were unchanged in neurons with each of three *PSEN1* mutations (M146I, Y115C, and Intron 4). However, levels of the lysosomal protein LAMP1 were increased in both *APP* and *PSEN1* Y115C or M146I mutant neurons (Figures 1D and 1F), indicating that both classes of mutations share defects in late endosomes and/or lysosomes.

### Accumulation of Enlarged Late Endosomes and Lysosomes in Human Neurons with *PSEN1* or *APP* Mutations

**Figure 2:**
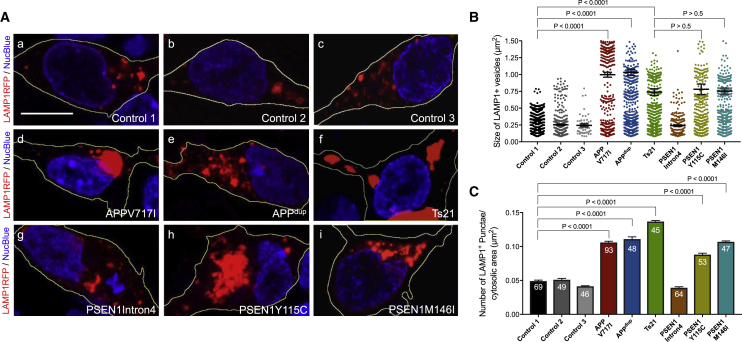
Accumulation of Enlarged Late Endosomes and Lysosomes in Human Neurons with *PSEN1* or *APP* Mutations (A) Representative images of neurons expressing a LAMP1-RFP fusion protein (red; blue, nuclei labeled with NucBlue). Scale bar, 10 μm. (B and C) Significant increases in the size (B) and number per unit area (C) of LAMP1-RFP-positive late endosomes and/or lysosomes in *APP* and *PSEN1* mutant (Y115C, M146I) neurons, but not *PSEN1* Intron 4 neurons, compared with non-demented controls (one-way ANOVA with Tukey correction between controls and AD neurons in each case). LAMP1-RFP^+^ vesicle size measures in (B) were from three independent experiments, including an independent neural induction in each case. Total number of vesicles measured (n): 484 (Control 1), 341 (Control 2), 50 (Control 3), 199 (*APP* V717I), 561 (*APPdup*), 579 (*Ts21*), 187 (*PSEN1* Intron4), 321 (*PSEN1* Y115C), 358 (*PSEN1* M146I). Number of vesicles per unit cytoplasmic area (C) was measured in three independent experiments (n = 3), with total numbers of cells indicated within each bar. Error bars, SEM. See also Figure S2.

To further investigate potential defects in the lysosomal system, we studied late endosomes and/or lysosomes in human cortical neurons with mutations in *APP* and *PSEN1* by live imaging. The number and size of lysosomes was increased in monogenic AD neurons of each genotype *(APP* and *PSEN1* mutations, Ts21), with the exception of *PSEN1 Intron 4* neurons (Figures 2A–2C). Neurons with *APP* or *PSEN1* mutations had accumulations of clustered lysosomes in their cell bodies (Figure 2A). In addition, the average size (area) of lysosomes in *APP* and *PSEN1* mutant neurons was significantly larger than that of control neurons (Figure 2B). This difference in average lysosome size was due to a change in the distribution of lysosome sizes within mutant neurons, whereby mutant neurons contained a population of very large lysosomes, together with a set of lysosomes of the same size as those in control neurons (Figure 2B). To confirm that LAMP1 expression does not alter lysosome size, we examined the size of LAMP1^+^ vesicles by either expression of RFP-tagged LAMP1 (live cell) or immunostaining for endogenous LAMP1 (fixed cell) in control neurons. We observed a very similar size distribution profile using either method (Figures S2A and S2B). Furthermore, we analyzed the size of endogenous LAMP1^+^ vesicles in control and *APP* V717I neurons. Consistent with our data using RFP-tagged LAMP1, we observed a significant increase in the size of endogenous LAMP1 in *APP* mutant neurons compared with control (Figures S2C and S2D).

The lack of detectable differences in lysosomal biology at this early stage in *PSEN1 Intron4* neurons is surprising, given that these neurons have similar changes in Aβ peptide production as the other *PSEN1* mutant neurons (Figure S1). However, the molecular nature of the *PSEN1 Intron4* mutation is qualitatively different from the majority of *PSEN1* mutations, which are missense protein-coding mutations that result in single amino acid substitutions (De Jonghe et al., 1999), and may enable some degree of compensation by the more abundant wild-type form of PSEN1 at these stages in relatively young neurons.

### Impaired Axonal Lysosome Transport and Proteolysis Deficits in Human *APP* and *PSEN1* Mutant Neurons

Given the accumulation of abnormally large lysosomes in the cell bodies of *APP* and *PSEN1* mutant neurons, we measured axonal transport of lysosomes in human familial AD neurons and controls. Axonal trafficking of lysosomes was quantified using time-lapse microscopy of axons in a microfluidic device, with lysosomes labeled with a fluorescent dye that preferentially identifies acidic organelles (LysoTracker). We observed that LysoTracker-positive vesicles were concentrated near the somatodendritic region (Figure S3). Furthermore, to prove that LysoTracker-positive structures are functional lysosomes, we treated LysoTracker-stained neurons with glycyl-L-phenylalanine-β-naphthylamide (GPN), which is a substrate of lysosomal protease cathepsin C that triggers osmotic lysis of lysosomes after cleavage. We observed that cleavage of GPN by cathepsin C diminished fluorescence of LysoTracker-positive organelles, confirming that they are lysosomes (Figure S4). For this and subsequent analyses, the *PSEN1 Intron4* mutation was not included, given the lack of endosome, lysosome, and autophagy phenotypes.

**Figure 3:**
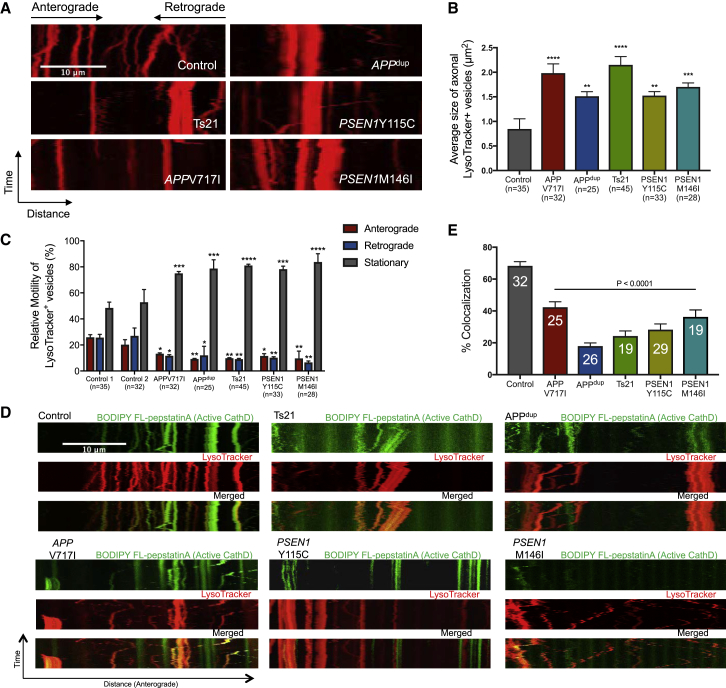
Impaired Axonal Lysosome Transport and Proteolysis Deficits in Human *APP* and *PSEN1* Mutant Neurons (A) Representative kymographs showing transport of lysosomes labeled with LysoTracker Red DND 99. Scale bar, 10 μm. (B) Significant increase in the average size of axonal lysosome in both *APP* and *PSEN1* mutant (Y115C, M146I) neurons, compared with non-demented control (one-way ANOVA with Tukey correction between control and AD neurons in each case). Total number of axons measured (n): 35 (Control), 32 (*APP* V717I), 25 (*APPdup*), 45 (*Ts21*), 33 (*PSEN1* Y115C), and 28 (*PSEN1* M146I). Error bars, SEM. (C) Significant reduction in lysosome motility in both anterograde and retrograde directions in both *APP* and *PSEN1* mutant neurons, compared with controls (one-way ANOVA with Tukey correction between controls and AD neurons in each case). Total number of axons measured (n): 35 (Control 1), 32 (Control 2), 32 (*APP* V717I), 25 (*APPdup*), 45 (*Ts21*), 33 (*PSEN1* Y115C), and 28 (*PSEN1* M146I). Error bars, SEM. (D) Proteolysis deficits in both *APP* and *PSEN1* mutant (Y115C, M146I) neurons, as detected by live imaging of iPSC-derived cortical neurons co-labeled with 100 nM LysoTracker Red DND-99 and BODIPY-FL-pepstatinA. Scale bar, 10 μm. (E) Significant reduction in the percentage of lysosomes that contained CatD-positive signals in both *APP* and *PSEN1* mutant (Y115C, M146I) neurons, compared with non-demented controls (one-way ANOVA with Tukey correction between controls and AD neurons in each case). Axons were measured in three independent experiments, with total numbers of axons (n) indicated within each bar. Error bars, SEM. See also Figures S3 and S4.

Consistent with previous reports of similar assays (Hung and Coleman, 2016), lysosomal movements were observed in both anterograde and retrograde directions in these neurons (Figure 3A). Moreover, the average size of axonal lysosomes was significantly increased in *APP* or *PSEN1* (M146I and Y115C) mutant neurons (Figure 3B). Analyses of the rates of lysosomal transport revealed that *APP* or *PSEN1* (M146I and Y115C) mutations reduced the numbers of moving lysosomes (Figure 3C). Furthermore, this reduction was seen in both anterograde and retrograde directions (Figure 3C).

Lysosomes accumulating within the distal ends of swollen axons in mouse models of AD contained low levels of multiple luminal lysosomal proteases (Gowrishankar et al., 2015), including cathepsin D (CatD). To investigate CatD activation within lysosomes in control and monogenic AD neurons, we live-imaged axons co-labeled with LysoTracker and BODIPY-FL-pepstatin A, which binds selectively to active CatD (Chen et al., 2000). To analyze the extent of co-migration between lysosomes and activated CatD, kymographs were generated for each channel (GFP and RFP) and for their overlays (Figure 3D). We then quantified the extent of co-migration in the axon by comparing pairs of kymographs generated for each channel (Figure 3E). In control neurons, over 60% of the lysosomes contained activated CatD (Figure 3E). However, fewer than 40% of the lysosomes in monogenic AD neurons contained activated CatD (Figure 3E).

### Defective Degradation of Autophagosomes in Human *APP* and *PSEN1* Mutant Neurons

**Figure 4:**
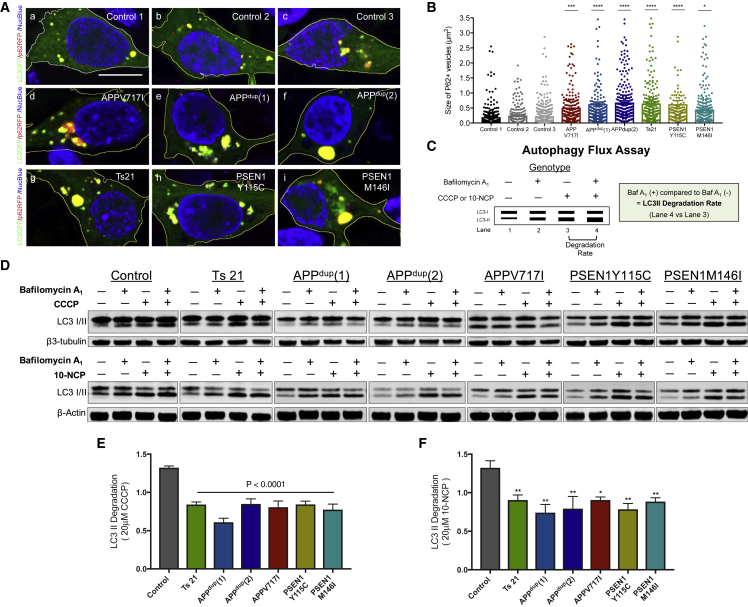
Defective Degradation of Autophagosomes in Human *APP* and *PSEN1* Mutant Neurons (A) Increased size of autophagosomes in human cortical excitatory neurons with *APP* and *PSEN1* mutations, as detected by live imaging of iPSC-derived neurons co-expressing p62-RFP (red) and a LC3-GFP (green) fusion proteins (blue, nuclei labeled with NucBlue). Scale bar, 10 μm. (B) Significant increases in the size of p62-RFP-positive autophagosomes in both *APP* and *PSEN1* mutant (Y115C, M146I) neurons, compared with non-demented controls (one-way ANOVA with Tukey correction between controls and AD neurons in each case). p62-RFP^+^ vesicle size measures in (B) were from three independent experiments, including an independent neural induction in each case. Total number of vesicles measured (n): 324 (Control 1), 183 (Control 2), 448 (Control 3), 376 (*APP* V717I), 355 (*APPdup*) (1), 350 (*APPdup*) (2), 372 (*Ts21*), 225 (*PSEN1* Y115C), and 399 (*PSEN1* M146I). Error bars, SEM. (C) Diagram of the autophagy flux assay. Autophagic degradation was calculated by comparing LC3-II levels following treatment with autophagy activators (CCCP or 10-NCP) in the presence (lane 4) or absence (lane 3) of bafilomycin A1 (i.e., lane 4 versus lane 3). (D–F) Autophagosome degradation was significantly reduced in both *APP* and *PSEN1* mutant (Y115C or M146I) neurons, compared with non-demented controls, as calculated from the western blot analysis (two-tailed t test between controls and AD neurons). Representative western blots of LC3I/II and neuron-specific β3-tubulin from neurons derived from three independent non-demented controls and monogenic AD iPSCs are shown (D). Autophagosome degradation following autophagy induction with either CCCP (20 μM) (E) or 10-NCP (20 μM) (F) in the absence or presence of bafilomycin A1 (200 nM) was calculated from four independent experiments (n = 4), including an independent neural induction in each case. Error bars, SEM.

Impaired lysosomal clearance can be associated with additional changes in autophagy, including accumulation of autophagic vesicles (Lie and Nixon, 2018). Therefore, we carried out a number of studies of autophagy in human familial AD neurons. To assess autophagosome size, we expressed an RFP-fusion of the autophagy receptor p62/SQSTM1 in neurons of each genotype and measured the size of p62/SQSTM1-positive autophagic vesicles in control and monogenic AD neurons (Figure 4A). We observed a striking accumulation of enlarged p62-positive autophagosomes in both *APP* and *PSEN1* mutant neurons compared to control neurons, indicating impairment in autophagy (Figures 4A and 4B).

Given the dynamic nature of autophagy, we measured autophagic flux in control and monogenic AD neurons to quantify autophagosome synthesis and degradation, using a previously reported quantitative approach (Figure 4C) (Rubinsztein et al., 2009). To do so, we treated neurons with the mitochondrial respiratory chain uncoupler (CCCP) to activate autophagy by triggering the loss of mitochondrial membrane potential (Figure 4C). The degradative phase of autophagy was blocked by v-ATPase inhibition with bafilomycin A1 (Baf A_1_) in either the presence or absence of autophagy induction by CCCP (Figure 4C). Western blot analysis of the relative amounts of LC3-I and LC3-II among the different conditions (with and without CCCP, with and without Baf A_1_) enabled the measurement of both the synthetic and degradative phases of autophagy (Figure 4C) (Rubinsztein et al., 2009).

Autophagy was induced in both control and monogenic AD neurons by CCCP treatment, as demonstrated by significant upregulation of LC3-II in the presence of CCCP and Baf A_1_ compared with neurons treated with Baf A_1_ alone (Figure 4D). However, comparing neurons treated with CCCP to induce autophagy with those treated with CCCP and Baf A_1_ (blocking autophagosome degradation) found a further elevation of LC3-II levels only in control neurons but not in the different AD neurons, indicating an impairment of autophagosome degradation in monogenic AD neurons (Figures 4D and 4E).

We confirmed that the degradative phase of autophagy is compromised in familial AD neurons using an alternative mTOR-independent autophagy inducer, the Akt inhibitor 10-(4′-(N-diethylamino) butyl)-2-chlorophenoxazine hydrochloride (10-NCP) (Tsvetkov et al., 2010). As seen with autophagy induction by mitochondrial stress with CCCP, autophagy induction with 10-NCP followed by inhibition of the vATPase with Baf A_1_ did not result in a further increase in LC3-II levels in familial neurons (Figures 4D and 4F). As with CCCP-induced autophagy, familial AD neurons demonstrated defective autophagy degradation when autophagy was induced by Akt inhibition (Figures 4D and 4F).

### Gamma-Secretase Inhibition Disrupts Function of the Lysosomal-Autophagic System in Human Neurons

Previous studies have suggested that intraneuronal accumulation of the C terminus of APP (APP-CTF) is a consequence of impaired lysosomal-autophagic degradation, rather than a cause of lysosome or autophagy dysfunction (Peric and Annaert, 2015). Processing of APP by BACE1 in the endosome generates the 99 amino acid C-terminal fragment of APP (β-CTF), which is in turn a substrate for γ-secretase in the lysosome.

**Figure 5:**
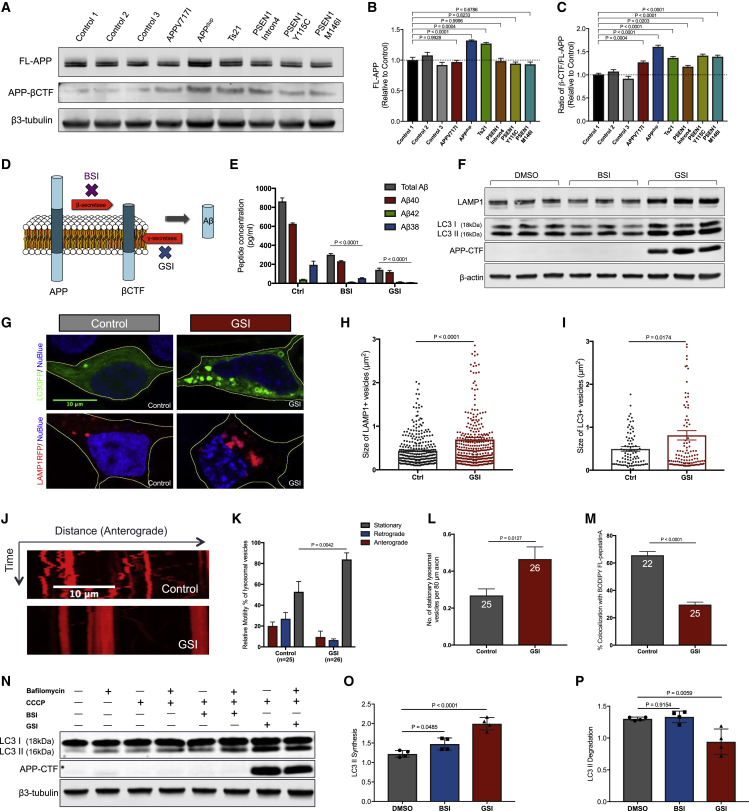
Gamma-Secretase Inhibition Disrupts Function of the Lysosomal-Autophagic System in Human Neurons (A–C) APP-β-CTF levels are significantly increased in human cortical excitatory neurons with *APP* and *PSEN1* mutations (intron 4, Y115C, or M146I), compared with non-demented controls (two-tailed t test between controls and AD neurons). Representative western blots of FL-APP, APP-βCTF, and neuron-specific β3-tubulin in three independent control and AD neurons are shown (A). Levels of FL-APP (B) and ratio of βCTF/FL-APP (C) were calculated relative to β3-tubulin and normalized to non-demented controls (n = 3). Error bars, SEM. (D) Schematic of APP processing to generate Aβ peptides. (E and F) Multiplexed ELISA (MSD) quantification of extracellular Aβ peptides (E) from non-demented control neurons following treatment with BSI shows significant reduction in the production of Aβ38, Aβ40, and Aβ42, compared to Ctrl. Similarly, GSI significantly reduces extracellular Aβ. Representative western blots of LAMP1, LC3I/II, APP-CTF, and neuron-specific β3-tubulin are shown (F). Neurons treated from day 65 to day 85 with the indicated compounds at a final concentration of 5 μM. Data were calculated from three independent experiments (n = 3), including an independent neural induction in each case. Error bars, SEM. (G) Live imaging of iPSC-derived neurons expressing LAMP1-RFP (red; blue, nuclei labeled with NucBlue) or LC3-GFP fusion protein (green; blue, nuclei labeled with NucBlue). Scale bar, 10 μm. (H and I) Significant increases in the size of (H) LAMP1-RFP-positive late endosomes and/or lysosomes and (I) LC3-GFP-positive autophagosomes in non-demented control neurons treated with GSI (two-tailed t test between DMSO and GSI-treated neurons in each case). LAMP1-RFP^+^ and LC3-GFP^+^ vesicle size measures in (H) or (I) were from three independent experiments, including an independent neural induction in each case. Total number of vesicles measured in (H) (n): 326 (Ctrl), 350 (GSI). Total number of vesicles measured in (I) (n): 90 (Ctrl), 111 (GSI). Error bars, SEM. (J) Impaired axonal lysosome transport in non-demented control human neurons treated with GSI, as detected by live imaging of iPSC-derived neuron labeled with 100 nM LysoTracker Red DND-99. Scale bar, 10 μm. (K and L) Significant reduction in lysosome motility (K) and number of stationary lysosomal vesicles (L) in non-demented control human neurons treated with GSI (two-tailed t test between DMSO and GSI-treated neurons in each case). Total number of axons measured in (H) (n): 25 (Ctrl), 26 (GSI). Axons were measured in three independent experiments, with total numbers of axons (n) indicated within each bar in (L). Error bars, SEM. (M) Significant reduction in the percentage of lysosomes that contained CatD-positive signals in non-demented control human neurons treated with GSI (two-tailed t test between DMSO and GSI-treated neurons in each case). Axons were measured in three independent experiments, with total numbers of axons (n) indicated within each bar. Error bars, SEM. (N–P) Autophagosome degradation was significantly reduced in non-demented control human neurons treated with GSI, as detected by western blot analysis (two-tailed t test between DMSO and GSI-treated neurons). Representative western blots of LC3I/II and neuron-specific β3-tubulin in non-demented control human neurons treated with DMSO, BSI, or GSI are shown (N). Rates of autophagosome (O) synthesis and (P) degradation following autophagy induction with CCCP (20 μM) in the absence or presence of bafilomycin A1 (200 nM) were calculated relative to β3-tubulin from four independent experiments (n = 4). Error bars, SEM.

We observed a significant increase in APP-β-CTF levels in both *APP* and *PSEN1* mutant neurons compared to control neurons (Figures 5A–5C). To further investigate the consequences of accumulation of β-CTF on the lysosomal and autophagy systems in non-diseased control neurons, we used small molecule inhibitors of γ-secretase to prevent turnover of β-CTF (Figure 5D). As expected, inhibition of either γ- or β-secretase significantly reduced extracellular Aβ peptides (Figure 5E), and γ-secretase inhibition led to a marked accumulation of β-CTF (Figure 5F).

Inhibition of γ-secretase led to an increase in both LAMP1 protein and LC3-II levels in healthy control neurons (Figure 5F). Together with the increase in LAMP1 protein levels, there was a significant increase in the number of enlarged lysosomes in neurons following γ-secretase inhibition (Figures 5G and 5H). Furthermore, γ-secretase inhibition led to an accumulation of LC3-positive autophagosomes, with γ-secretase inhibition markedly increasing the number and size of the GFP-LC3 puncta in neurons (Figures 5G and 5I). In addition, live imaging of axonal transport of lysosomes found that lysosome transport was significantly impaired in neurons following γ-secretase inhibition (Figures 5J–5L). Inhibition of γ-secretase led to both an increase in stationary lysosomes within axons, as well as a reduction in both retrograde and anterograde lysosome transport (Figures 5J–5L). Furthermore, we observed a significant reduction in the percentage of lysosomes that contained CatD-positive signals in healthy control neurons treated with GSI (Figure 5M).

To investigate whether the observed autophagosome accumulation was caused by induction of autophagy or by inhibition of degradation, we measured autophagic flux as described above (Figure 4C). In contrast with familial AD neurons, we found that autophagosome synthesis was increased following either β-secretase or γ-secretase inhibition (Figures 5N and 5O). However, whereas degradation was not changed following β-secretase inhibition, γ-secretase inhibition significantly reduced autophagosome degradation (Figure 5P). These data indicate that reducing γ-secretase turnover of C terminus of APP leads to lysosomal-autophagic dysfunction, suggesting that the turnover of the C terminus of APP by γ-secretase has a regulatory role in modulating the lysosomal-autophagy system.

### Dysfunction in the Lysosome-Autophagy System in Familial AD Neurons Is Reversed by β-Secretase Inhibition

Given that inhibition of APP-CTF turnover by γ-secretase negatively impacted the lysosomal-autophagy system in healthy neurons, we investigated the effect of manipulating APP processing on lysosome-autophagy defects in AD neurons. We focused on addressing whether reducing the production of β-CTF by inhibition of β-secretase would ameliorate autophagy defects in monogenic AD neurons (*PSEN1* and *APP* mutations, trisomy 21).

**Figure 6:**
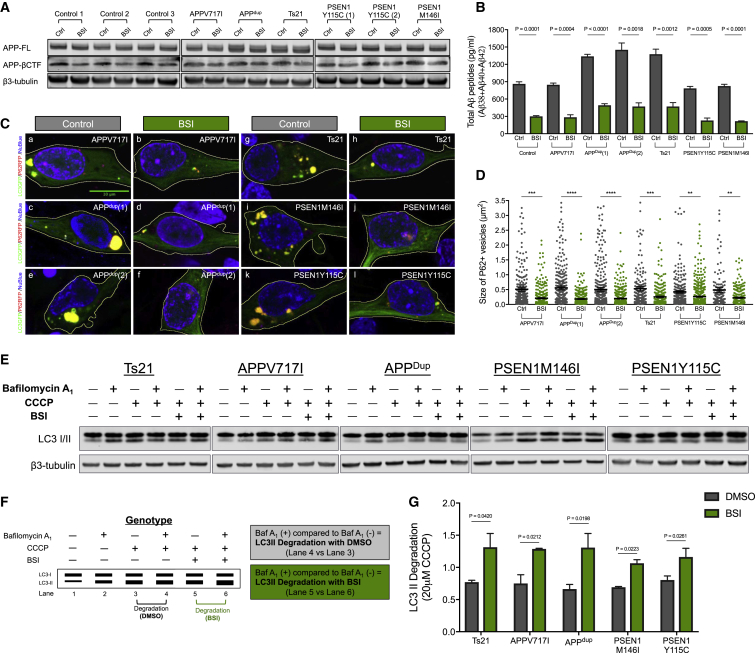
Dysfunction in the Lysosome-Autophagy System in Familial AD Neurons Is Reversed by β-Secretase Inhibition (A) BSI reduces APP-βCTF level in all genotypes. Representative western blots of FL-APP, APP-βCTF, and neuron-specific β3-tubulin are shown. (B) Multiplexed ELISA quantification of extracellular Aβ peptides from monogenic AD neurons following treatment with BSI shows significant reduction in the production of total Aβ peptides, compared to controls (two-tailed t test between DMSO and BSI-treated neurons in each case). Neurons treated between from day 65 to day 85 with BSI at a final concentration of 5 μM. Data were calculated from three independent experiments (n = 3), including an independent neural induction in each case. Error bars, SEM. (C) Live imaging of iPSC-derived neurons co-expressing p62-RFP (red) and LC3-GFP (green) fusion proteins (blue, nuclei labeled with NucBlue). Scale bar, 10 μm. (D) Significant reductions in the size of p62-RFP-positive autophagosomes in monogenic AD neurons treated with BSI, compared with DMSO-treated controls (two-tailed t test between DMSO and BSI-treated neurons in each case). p62-RFP^+^ vesicle size measures in (C) were from three independent experiments, including an independent neural induction in each case. Total number of vesicles measured (n): 558 (*APP* V717I Ctrl), 396 (*APP* V717I BSI), 479 (*APPdup*(*1*) Ctrl), 484 (*APPdup*(*1*) BSI), 574 (*APPdup*(*2*) Ctrl), 541 (*APPdup*(*2*) BSI), 384 (*Ts21* Ctrl), 399 (*Ts21* BSI), 628 (*PSEN1* Y115C Ctrl), 614 (*PSEN1* Y115C BSI), 240 (*PSEN1* M146I Ctrl), and 291 (*PSEN1* M146I BSI). Error bars, SEM. (E) Representative western blots of LC3I/II and neuron-specific β3-tubulin in familial AD neurons treated with DMSO or BSI are shown. (F and G) Schematic of the autophagy flux assays (F) used for this set of comparisons (E). Autophagosome degradation (basal level) was calculated by comparing LC3-II levels following treatment with CCCP in the presence (lane 4) or absence (lane 3) of bafilomycin A1 (i.e., lane 4 versus lane 3). Similarly, autophagosome degradation (treated with BSI) was calculated by comparing LC3-II levels following treatment with CCCP in the presence (lane 5) or absence (lane 6) of bafilomycin A1 (i.e., lane 5 versus lane 6). (G) LC3-II levels were calculated relative to β3-tubulin from three independent experiments (n = 3) (two-tailed t test between DMSO and BSI-treated neurons in each case). Error bars, SEM. See also Figure S5.

As expected, inhibition of β-secretase activity significantly reduced the production of total APP-βCTF levels (Figure 6A) and total extracellular Aβ peptides in neurons of all genotypes (Figure 6B). We observed that manipulation of β-secretase activity alters LAMP1 expression in genetic forms of AD (Figure S5). In addition, there was a significant reduction in the size of p62/SQSTM1-positive vesicles in familial AD and trisomy 21 neurons, suggesting that β-secretase inhibition corrected defects in the degradative phase of autophagy (Figures 6C and 6D). Measurements of autophagic flux in monogenic AD neurons treated with β-secretase inhibitor were performed using the same methods as described above. As indicated by the imaging of p62-RFP, β-secretase inhibition led to a significant increase of the degradation phase of autophagy compared to vehicle-treated neurons of each genotype (Figures 6E–6G).

### Autophagy Defects in *PSEN1* Mutant Neurons Are Rescued by CRISPR Knockout of *APP*

**Figure 7:**
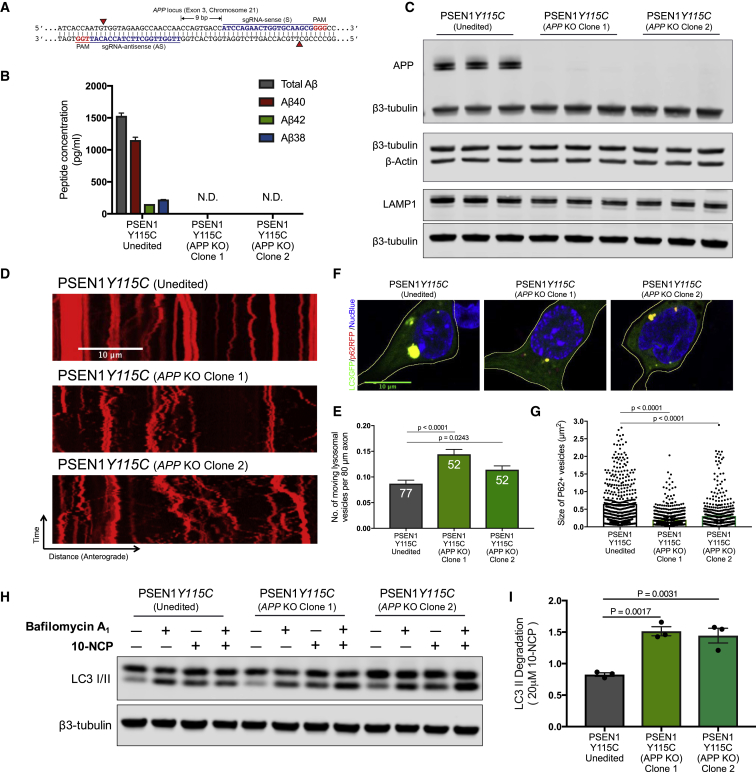
Autophagy Defects in *PSEN1* Mutant Neurons Are Rescued by CRISPR Knockout of *APP* (A) Double nickase strategy with sense (S) and antisense (AS) single guide RNAs (sgRNAs) separated by 9 bp at exon 3 of the *APP* locus on chromosome 21. Red arrows indicate the cut sites of Cas9^D10A^ nickase, which generates 5′ overhangs at target sites. (B) CRISPR targeting of both alleles of *APP* was confirmed by the complete absence of extracellular Aβ peptides generated by *PSEN1* Y115C;*APP* knockout (KO) neurons, compared with isogenic *PSEN1* Y115C neurons. Multiplexed ELISA quantification of extracellular Aβ peptides from unedited and two independent clones of *APP* KO neurons from three independent experiments (n = 3). (C) APP protein was not detected in two independent clones of *PSEN1* Y115C; *APP* KO neurons, confirming complete knockout of both *APP* alleles. In contrast, total LAMP1 levels are decreased in two independent clones of *PSEN1* Y115C;*APP* KO neurons (90 days post-neural induction), compared with unedited *PSEN1* Y115C neurons (two-tailed t test between unedited and KO neurons). Representative western blots of APP, LAMP1, β-actin, and neuron-specific β3-tubulin unedited isogenic *PSEN1* Y115C and two independent clones of *PSEN1* Y115C;*APP* KO neurons. (D and E) Live imaging of iPSC-derived neurons labeled with 100 nM LysoTracker Red DND-99 (D). Scale bar, 10 μm. Significant increases in the number of moving lysosomal vesicles (E) in two independent clones of *PSEN1* Y115C;*APP* KO neurons, compared with unedited isogenic *PSEN1* Y115C neurons (two-tailed t test between unedited and KO neurons). Axons were measured in three independent experiments, with total numbers of axons (n) indicated within each bar. Error bars, SEM. (F and G) Significant decreases in the size of p62-RFP-positive autophagosomes (F) in two independent clones of *PSEN1* Y115C;*APP* KO neurons, compared with unedited isogenic neurons (two-tailed t test between unedited and KO neurons) (G). p62-RFP^+^ vesicle size measures in (G) were from three independent experiments, including an independent neural induction in each case. Total number of vesicles measured (n): 799 (*Unedited;PSEN1* Y115C), 840 (*PSEN1* Y115C;*APP* KO Clone 1), and 602 (*PSEN1* Y115C;*APP* KO Clone 2). Error bars, SEM. (H and I) Impaired autophagosome degradation was significantly rescued in two independent clones of *PSEN1* Y115C;*APP* KO neurons, compared with unedited isogenic neurons (two-tailed t test between unedited and KO neurons). Representative western blots of LC3I/II and neuron-specific β3-tubulin in two independent clones of *PSEN1* Y115C;*APP* KO neurons are shown (H). (I) LC3-II levels following autophagy induction with 10-NCP (20 μM) in the absence or presence of bafilomycin A1 (200 nM) were calculated relative to β3-tubulin from three independent experiments (n = 3). Error bars, SEM.

While the pharmacological manipulation of both BACE1 and γ-secretase strongly implicated APP in mediating PSEN1-dependent phenotypes, they do not formally prove this. To investigate this, we used CRISPR genome engineering to knockout both alleles of *APP* in *PSEN1* Y115C iPSCs (Figure 7A). Cortical neurons generated from two independent clones of *PSEN1* Y115C*;APP*^*−/−*^ iPSCs had no detectable production of extracellular Aβ peptides (Figures 7B and 7C), confirming the complete absence of APP.

Deletion of *APP* in *PSEN1* Y115C neurons reduced LAMP1 protein levels (Figure 7C) and increased the axonal transport of lysosomes (Figures 7D and 7E), compared with isogenic *PSEN1* Y115C neurons. Removal of *APP* also rescued autophagy defects in *PSEN1* Y115C neurons. There was a significant reduction in the size of p62-RFP positive vesicles in the APP knockout neurons, compared with isogenic *PSEN1* Y115C neurons (Figures 7F and 7G). Measuring autophagic flux in isogenic *PSEN1* Y115C and *PSEN1* Y115C*;APP*^*−/−*^ neurons demonstrated that removal of APP protein rescued the impairment in the degradative phase of autophagy seen in *PSEN1* Y115C neurons (Figures 7H and 7I). Therefore, these data demonstrate that the lysosomal-autophagy defects observed in *PSEN1* mutant neurons are *APP*-dependent.

## Discussion

We report here that mutations in *APP* and *PSEN1* that are causal for early onset AD lead to major defects in lysosome function and autophagy in human neurons. The rapid appearance of these defects indicates that they are a direct effect of those mutations, and not a secondary consequence of a primary degenerative process, such as protein aggregation. Consistent with this, we find that inhibition of γ-secretase causes lysosomal and autophagy dysfunction in healthy human neurons. *APP* and *PSEN1* mutant neurons accumulate APP-β-CTF and reducing production of C-terminal APP by inhibition of BACE1 rescued lysosome-autophagy phenotypes in both *APP* and *PSEN1* mutant neurons. Defects in the lysosome and autophagy systems due to *PSEN1* mutations are prevented by CRISPR knockout of *APP*, demonstrating that lysosome and autophagy dysfunction in *PSEN1* mutant neurons are dependent on the presence of APP. These data demonstrate that compromised proteolysis of the C-terminal of APP by γ-secretase by autosomal dominant mutations in *PSEN1* and *APP* causes dysfunction of the lysosomal and autophagy systems in human neurons and represents a potential driver of neurodegeneration in monogenic AD.

We and others have previously reported that neurons generated from iPSCs with genetic forms of AD recapitulate aspects of the disease, including increased Aβ peptide production in trisomy 21 and *APP* duplication neurons (Moore et al., 2015, Israel et al., 2012) and increased production of longer forms of Aβ in *PSEN1* and *APP* mutant neurons (Moore et al., 2015, Muratore et al., 2014, Woodruff et al., 2016, Israel et al., 2012). We now report that AD mutations in *APP* and *PSEN1* converge on disruption of the lysosome and autophagy system in human neurons. Given growing evidence from genome-wide association studies implicating the endolysosomal and autophagy systems in AD risk (Karch and Goate, 2015), these data suggest that dysfunction in these systems may represent an early pathogenic process in the disease.

The contributions of the endolysosomal and autophagy systems to AD initiation and progression are currently unclear, with debate surrounding whether observed defects in those systems are causes or consequences of neuronal dysfunction and neurodegeneration (Nixon, 2017). Post-mortem analyses of the cerebral cortex have demonstrated enlarged early endosomes in neurons of individuals with trisomy 21 or *APP* duplication (Cataldo et al., 2000), as well as in transgenic mouse models expressing human *APP*, trisomy 21 fibroblasts, and various cell lines overexpressing different forms of APP (Nixon, 2017). Focal axonal swellings containing accumulations of lysosomal dense bodies and autophagic vacuoles have been observed in post-mortem brain samples of Alzheimer’s patients (Nixon, 2013, Nixon, 2017, Nixon et al., 2005), suggesting a failure of their axonal transport.

We have found that mutations in *APP* and *PSEN1* converge on defects in lysosome function and autophagy. The presence of these changes within relatively young neurons, combined with the ability to induce these phenotypes by acute gamma-secretase inhibition, indicates that these defects are a direct consequence of mutations in *APP* and *PSEN1*, and not an indirect effect of another degenerative process, such as protein aggregation. *PSEN1* mutant neurons do not have early endosome defects, in contrast with *APP* mutant neurons. This is consistent with previous analysis from post-mortem studies showing that early endosomal abnormalities were not found in individuals with either *PSEN1* or *PSEN2* mutations, despite Aβ overproduction and amyloid plaque deposition (Cataldo et al., 2000). In addition, endosomal dysfunction was also absent in both primary fibroblasts from patients with *PSEN1* mutation and neurons from PS1 and βAPP transgenic mice (Cataldo et al., 2000). Our finding that the different *APP* and *PSEN1* mutations overlap in lysosome and autophagosome dysfunction suggest that the primary site of action of these mutant proteins is the lysosome, with endosomal changes being a secondary effect.

Processing of APP by BACE1 in the endosome generates the APP C-terminal fragment (APP-β–CTF), which is in turn a substrate for γ-secretase in the lysosome. The role of APP-CTF in AD pathogenesis is the subject of considerable debate. Previous studies have suggested that intraneuronal accumulation of APP-CTF is a consequence of impaired lysosomal-autophagic degradation, rather than a cause of endolysosome or autophagy dysfunction (Peric and Annaert, 2015). In addition, a recent study showed that deletion of *APP* expression in mice deficient for all γ-secretases, by deletion of the genes encoding Aph1 subunits, failed to rescue progressive neurodegeneration (Acx et al., 2017). In that case, there was a general accumulation of many γ-secretase substrates, due to complete loss of γ-secretase function, which is in contrast to the hypomorphic character of autosomal dominant pathogenic mutations in *PSEN1* (Moore et al., 2015, Chávez-Gutiérrez et al., 2012). Recent studies, however, have reported that intraneuronal aggregation of APP-CTF induced Aβ-independent lysosomal-autophagic pathology (Lauritzen et al., 2016, Jiang et al., 2016). We found that increasing intracellular APP-CTF by γ-secretase inhibition induced lysosomal-autophagic pathology: elevated levels of lysosomal LAMP1 and autophagosome LC3-II, increased density and size of LC3-positive puncta, and increased number of enlarged LAMP1-positive vesicles. In addition, γ-secretase inhibition markedly impaired axonal transport of lysosomes and led to autophagic defects.

Conversely, we find that dysfunction in lysosomal-autophagy system observed in human *APP* and *PSEN1* mutant neurons can be reversed by β-secretase inhibition, which reduces the supply of APP-β-CTF to the late endosome and/or lysosome (Jiang et al., 2010). These data argue that accumulation and/or altered turnover of APP-CTF by γ-secretase lead to dysfunction in the neuronal lysosomal-autophagy system. Importantly, the rescue of lysosomal-autophagy defects by CRISPR deletion of *APP* in a *PSEN1* mutant background formally demonstrates that those defects are dependent on APP protein, and not simply convergent independent phenotypes due to mutations in either gene.

The lack of detectable defects in lysosome and autophagosome function in *PSEN1 Intron4* neurons is surprising, given that these neurons have similar changes in Aβ peptide production as the other *PSEN1* mutant neurons. However, the molecular nature of the *PSEN1 Intron4* mutation is qualitatively different from the majority of *PSEN1* mutations, which are missense protein-coding mutations that result in single amino acid substitutions (De Jonghe et al., 1999). The *PSEN1 Intron4* mutation is a single base deletion within the splice donor site, which has been predicted to generate three different final mRNAs, two of which encode truncated forms of PSEN1, and the third includes an extra amino acid (De Jonghe et al., 1999). If this is the case in cortical neurons, the relative amounts of wild-type and full-length mutant PSEN1 proteins would be much less than 1:1, and relatively higher levels of the wild-type form of PSEN1 might enable short-term compensation for the deleterious effects of the mutant protein. Therefore, it will be essential to further study the effects of this mutation on lysosome and autophagosome function over longer time frames and in neurons homozygous for this mutation.

The early stage of axonal lysosome biogenesis is thought to begin in distal regions of axons with the merging of organelles derived from endocytic and autophagic pathways (Maday and Holzbaur, 2014). Further maturation of lysosomes requires retrograde transport to the cell soma where luminal proteases can be delivered from the trans-Golgi network efficiently (Maday et al., 2012). Such a mechanism allows neurons to effectively remove autophagic cargos from axon terminals and synapses. We found that AD mutations in *APP* and *PSEN1* markedly disrupted the axonal transport of lysosomes and those lysosomes accumulate within distal axons contained low levels of cathepsin D. This is similar to previous findings in human AD brain tissue as well as various AD mouse models in which focal axonal swellings containing accumulations of lysosomal dense bodies and autophagic vacuoles have been observed (Nixon, 2017).

Although autophagy activation may be a promising therapeutic strategy for AD, its beneficial role in disease pathogenesis is not clear. Several studies showed that increasing autophagy in mouse models of AD ameliorates amyloid pathologies and memory deficits (Spilman et al., 2010, Yang et al., 2011). However, autophagy induction has also been reported to activate the amyloidogenic pathway and increase Aβ secretion (Yu et al., 2005, Nilsson et al., 2013). Similarly, administration of rapamycin after the formation of amyloid plaques failed to rescue the cognitive deficits in mouse model of AD (Majumder et al., 2011). Our data suggest that induction of autophagy may become counterproductive in the face of impaired transport of axonal lysosomes, lysosomal clearance deficits and reduced autophagosome degradation, by further overburdening the already failing lysosomes and thus exacerbating autophagic accumulation in axons. Indeed, recent studies have demonstrated that restoring retrograde transport of autophagic vacuoles by overexpressing Sanpin proteins successfully reduced AD-associated autophagic stress (Tammineni et al., 2017). The present study on human cortical neurons provides further evidence that either reducing input of APP to the lysosomal-autophagy system, enhancing axonal transport or augmenting lysosome function at the early disease stages may represent potential therapeutic strategies to attenuate autophagic defects in AD. Overall, this study establishes a foundation for future investigation into cellular pathways enhancing autophagy and lysosomal proteolytic activity as an approach to ameliorating neurodegeneration in AD.

## STAR★Methods

### Key Resources Table

**Table:** 

REAGENT or RESOURCE	SOURCE	IDENTIFIER
**Antibodies**		

Mouse monoclonal anti-APP	BioLegend	Cat#802801; RRID: AB_2564648
Mouse anti-β-Amyloid, 1-16	BioLegend	Cat#803001; RRID: AB_2564653
Rabbit polyclonal anti-Tubulin β-3	BioLegend	Cat#802001; RRID: AB_2564645
Mouse monoclonal anti- β-actin	Sigma	Cat#A228; RRID: AB_476697
Rabbit polyclonal anti-LAMP1	abcam	Cat#;Ab62562 RRID:AB_2134489
Rabbit polyclonal anti-LC3B	Sigma	Cat#L7543; RRID: AB_796155
Chicken polyclonal anti-MAP2	abcam	Cat#Ab5392; RRID: AB_2138153

**Experimental Models: Cell Lines**		

Human: Non-demented control iPSC lines	Israel et al., 2012	N/A
Human: SFC840 iPSC lines	StemBANCC	N/A
Human: AD3.1 iPSC lines	StemBANCC	N/A
Human: PSEN1 Y115C iPSC lines	Moore et al., 2015	N/A
Human: PSEN1 M146I iPSC lines	Moore et al., 2015	N/A
Human: PSEN1 Intron4 iPSC lines	Moore et al., 2015	N/A
Human: APP V717I iPSC lines	Moore et al., 2015	N/A
Human: APP duplication iPSC lines	Israel et al., 2012	N/A
Human: Ts21 iPSC lines	Park et al., 2008	N/A

**Oligonucleotides**		

sgRNA-sense (S): ATCCAGAACTGGTGCAAGCGGGG	This paper	N/A
sgRNA-antisense (AS): TTGGTTGGCTTCTACCACATTGG	This paper	N/A

**Software and Algorithms**		

ImageJ	https://imagej.net/Welcome	RRID: SCR_003070
GraphPad Prism	https://www.graphpad.com/	RRID: SCR_002798

### Contact for Reagent and Resource Sharing

Further information and requests for resources and reagents should be directed to and will be fulfilled by the Lead Contact, Rick Livesey (r.livesey@ucl.ac.uk).

### Experimental Model and Subject Details

#### Human iPSC lines

Non-demented control iPSC lines: Non-Demented-Control (NDC) (Israel et al., 2012), SFC840 (StemBANCC), and AD3.1 (StemBANCC). All AD lines were previously reported and characterized: *PSEN1* Y115C, M146I, intron 4, and *APP* V717I iPSCs (Moore et al., 2015); *APP* duplication (Israel et al., 2012, Moore et al., 2015) and Ts21 iPSCs (Park et al., 2008, Moore et al., 2015, Shi et al., 2012b). This research was carried out in accordance with the UK Code of Practice for the Use of Human Stem Cell Lines.

### Method Details

#### Directed differentiation to human cortical neuron culture

Directed differentiation of iPSCs to cerebral cortex was carried out as described (Moore et al., 2015). Briefly, dissociated iPSCs were plated on GelTrex-coated 6-well plates and neural induction were initiated by changing into culture medium that supports neuronal differentiation and neurogenesis, a 1:1 mixture of N2- and B27-containing media (supplemented with dorsomorphin and SB431542 to inhibit TGF β signaling during neural induction). Neuroepithelial cells were harvested with dispase and replated in laminin-coated plates with FGF2-containing media. FGF2 was withdrew for 4 days to promote differentiation, passaged with accutase, and maintained for up to 120 days with a medium change every other day. To establish identity and quality of cortical neuronal inductions, gene expression profiling was performed on a custom gene expression panel. RNA was isolated from induced cortical neurons, 85 – 90 days after induction, using an RNA extraction kit (QIAGEN), according to the manufacturer’s instructions. Expression levels of mRNAs enriched in deep and upper layer cortical neurons were assessed on the Nanostring nCounter platform (Figure S1).

For drug treatments, all compounds were dissolved in DMSO at the concentrations specified, and DMSO was used as vehicle control in all experiments. Compounds were added every 48 hr during treatment period: β-secretase inhibitor OM99-2 (Calbiochem); and γ-secretase inhibitor, DAPT (Sigma).

#### Protein analysis

Extracellular Aβ 1-42, Aβ 1-40, and Aβ 1-38 were measured in conditioned media using multiplexed MesoScale Discovery assays on a Quickplex SQ120 instrument (MesoScale Discovery). All statistical analysis was performed between the entire set of controls samples and all samples of each genotype, using Student’s t test with the Bonferroni correction for multiple testing.

#### Immunoblotting

For immunoblotting, whole cell lysate protein was extracted with RIPA buffer (Sigma). Samples were separated on a 4%–12% SDS-PAGE and transferred to PVDF membranes. Proteins were detected (Li-Cor Odyssey system) by incubation with specific primary antibodies and appropriate secondary antibodies. Antibodies used in this study are listed in Key Resources Table.

#### Live cell imaging and confocal microscopy

For live cell imaging, Rab5A-GFP (Catalog no. C10586), LAMP1-RFP (Catalog no. C10597), LC3-GFP (Catalog no. P36235) and p62-RFP (Catalog no. P36241) were expressed in day 80-85 neurons using commercial viral vectors (ThermoFisher Scientific). Neurons were transferred into live-imaging solution (Invitrogen) 16 hours after infection in order to improve imaging of fluorescent proteins. Cells were viable and appeared morphologically normal in imaging medium for at least 24 hours. As an alternative to expression of LAMP1-RFP, neuronal lysosomes were visualized by incubation of 100 nM LysoTracker Red DND-99 (Molecular Probes) with or without the addition of Pepstatin A, BODIPY FL Conjugate (Invitrogen) in fresh medium for 30 minutes at 37°C. Neurons were washed and then replaced with fresh culture medium before imaging. For imaging axonal transport of lysosomes, neurons were cultured in microfluidic devices (XONA microfluidics) mounted onto glass coverslips coated with Geltrex (Life Technologies).

Time-lapse images were acquired using a Zeiss SP5 confocal microscope. Cultures were maintained at 37°C in a CO_2_ environment chamber. Time-lapse images were acquired in a single focal plane at 2 s intervals for 60 stacks. Data were collected in the axon, up to approximately 900 microns away from the cell body (assuming the cell body is located at the opening of the micro-channel). For the analysis of vesicle size of fluorescently labeled particles, the image was first pre-processed to reduce noise and the coordinates were calculated using differences of Gaussian, and then single particle was tracked with TrackMate plugin (Tinevez et al., 2017). ImageJ was used for generation of kymograph and analysis of particle motility as described previously (Hung and Coleman, 2016).

For immunostaining, cells were fixed in 4% paraformaldehyde (PFA) in PBS followed by permeabilization with Triton X-100 (Sigma). Fixed cells were blocked with 50% normal donkey serum (Sigma) in PBS, probed with primary antibodies diluted in blocking solution (antibodies listed in Key Resources Table) and detected with goat anti-mouse, anti-chicken or anti-rabbit secondary antibody coupled to Alexa Fluor 488 or 594. Confocal images were acquired using a Zeiss SP5 confocal microscope.

#### CRISPR-mediated gene knockout

The following sites were used for designing the optimal sgRNA pairs for nickase targeting and prediction of off-target sites: CRISPR Design (http://zlab.bio/guide-design-resources) and WTSI Genome Editing (https://www.sanger.ac.uk/htgt/wge/). The All-in-One nickase vector plasmid (gift from Prof S Jackson, Cambridge) (Chiang et al., 2016). Pairs of complementary DNA oligos were purchased in standard desalted format from IDT:

sgRNA-sense (S): 5′ ATCCAGAACTGGTGCAAGCGGGG 3′
sgRNA-antisense (AS): 5′ TTGGTTGGCTTCTACCACATTGG 3′

Both sense and antisense sgRNA oligos were cloned into the All-in-One nickase vector and the correct sequence was verified by Sanger sequencing. Plasmids were transfected into iPSCs by electroporation using the Neon Transfection System according to the manufacturer’s instructions (Life Technologies). Cells were trypsinised 48 hours after transfection, washed with Essential 8 medium (Thermo Fisher), and sorted based on EGFP signal into Geltrex (Life Technologies)-coated 10 cm^2^ dish (Thermo Scientific) at a low density and individual colonies were picked manually for clonal expansion.

### Quantification and Statistical Analysis

Unless otherwise specified, statistics analysis was performed using GraphPad Prism (Version 7). Unpaired Student’s t test was used to compare differences between two groups, assuming the data were normally distributed. One-way ANOVA with a post hoc Tukey test was used to analysis differences between more than two groups (i.e., genotypes). For precise p value calculation, a multiple t test was performed after ANOVA calculations. Significance threshold was defined as adjusted p value < 0.05. Error bars in all figures represent SEM. The number of biological replicates (n) is listed in the legend of each figure.

## Supplemental Information

Document S1. Figures S1–S5

Document S2. Article plus Supplemental Information
