# Supplementary material for: Altered γ-Secretase Processing of APP Disrupts Lysosome and Autophagosome Function in Monogenic Alzheimer’s Disease
Source: Cell Rep. 2018 Dec 26;25(13):3647–3660.e2. doi: 10.1016/j.celrep.2018.11.095 (PMC6315085; doi:10.1016/j.celrep.2018.11.095)
Supplement: Document S1. Figures S1–S5 [file mmc1.pdf]

Cell Reports, Volume *25*

Supplemental Information

Altered γ-Secretase Processing of APP Disrupts Lysosome and Autophagosome Function in Monogenic Alzheimer's Disease

Christy O.Y. Hung and Frederick J. Livesey

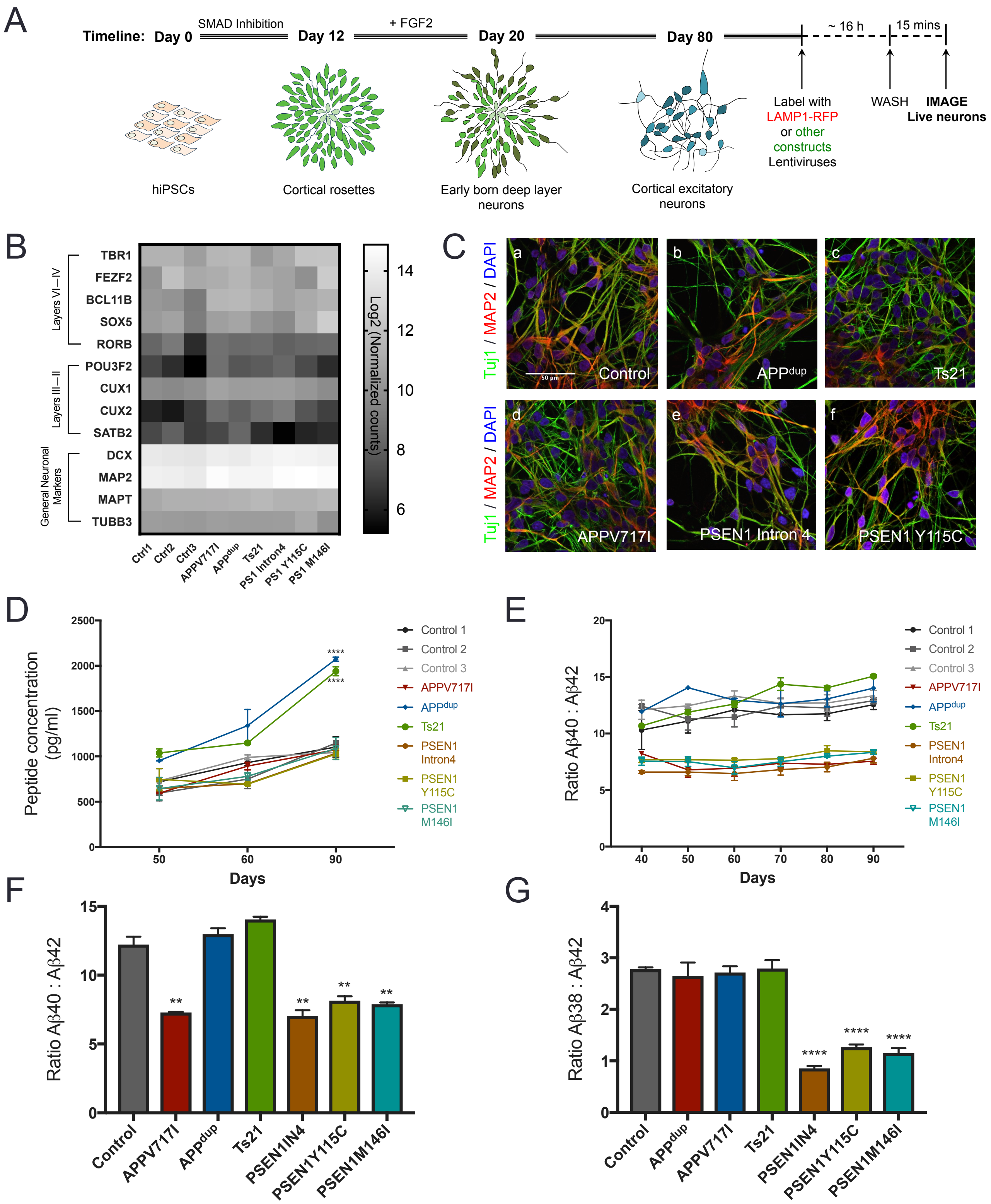

Figure S1. Characterisations of human cortical neurons. Related to Figure 1. (**A**) Schematic of the methods used here for generation of excitatory cortical neurons from human iPSCs. (**B**) Gene expression analysis of RNA, extracted at day 90 post-neuronal induction, using the Nanostring nCounter platform displayed on a log (base 2) scale, normalised to housekeeping genes. Transcripts associated with deep and upper layer cortical neurons were expressed by cultures of each genotypes from two independent neuronal inductions. (**C**) Representative immunohistochemistry of neurons generated from monogenic Alzheimer's disease (*APPdup*, *Ts21*, *APP*V717I, *PSEN1* intron 4, and *PSEN1* Y115C) iPSCs, expressing neuronal proteins (β3-tubulin, green and MAP2, red). (**D**) Neurons with *PSEN1* mutations generate equivalent amounts of total extracellular Aβ peptides over 50 days in culture compared with three different healthy control lines. *APP* V717I neurons also do not significantly alter the production of total Aβ peptides compared to controls. This is in contrast with *APP* (*dup*) or *Ts21* neurons, which significantly increase total Aβ peptide production. (**E**) *APP* V717I and *PSEN1* mutations have increased secretion of longer forms of Aβ relative to more abundant short forms, as reflected in significantly reduced Aβ40: Aβ42 ratios compared with non-demented control, Ts21 and *APP*(*dup*) neurons at all time points analysed (day 40 to day 90). In contrast, Ts21 and *APP*(*dup*) have an increase in production of all Aβ peptides, including Aβ42. (**F, G**) At day 80, *APP* V717I and *PSEN1* mutant neurons all have reduced Aβ40:Aβ42 ratios (F), but only *PSEN1* mutant neurons have a significant reduction in extracellular Aβ38:Aβ42, consistent with reduced γ-secretase processivity.

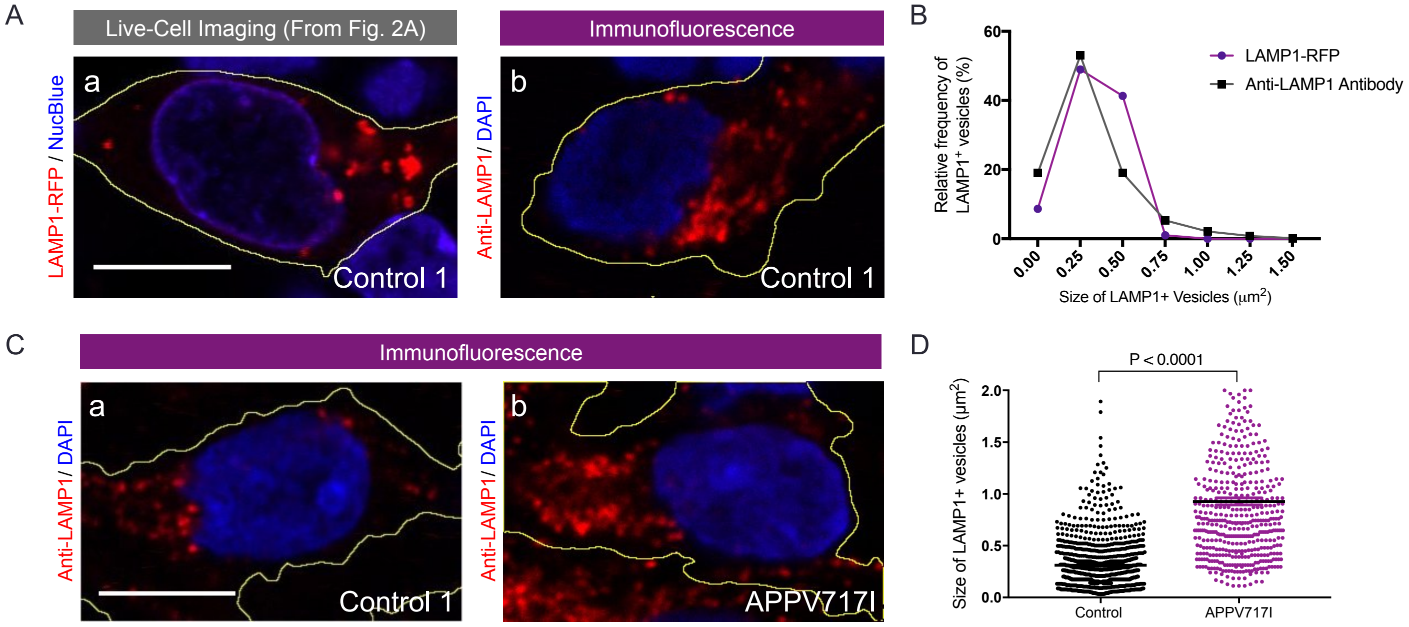

Figure S2. Size distribution profile. Related to Figure 2. (**A**) (a) Representative images of iPSC-derived neurons expressing LAMP1-RFP fusion protein (**reproduced from Figure 2A for comparison**) (red, LAMP1-RFP; blue, nuclei labelled with NucBlue). (b) Representative immunohistochemistry of iPSC-derived neurons expressing LAMP1 proteins (red, LAMP1; blue, DAPI). (**B**) Similar size distribution profile using either expression of RFP-tagged LAMP1 (live cell) or immunostaining for endogenous LAMP1 (fixed cell) in control neurons. Total number of vesicles measured (n): 484 (live cell) and 1087 (fixed cell). Error bars, S.E.M. (**C**) Representative immunohistochemistry of neurons generated from (a) healthy control line or (b) monogenic Alzheimer's disease (*APP*V717I) iPSCs, expressing LAMP1 protein (red, LAMP1 ; blue, DAPI). (**D**) A significant increase in the size of late endosomes/lysosomes in human cortical excitatory neurons with *APP* V717I mutation compared with non-demented control, as detected by immunohistochemistry of neurons expressing LAMP1 proteins (red, LAMP1 ; blue, DAPI). Total number of vesicles measured (n): 1088 (Control) and 485 (*APP* V717I). Error bars, S.E.M.

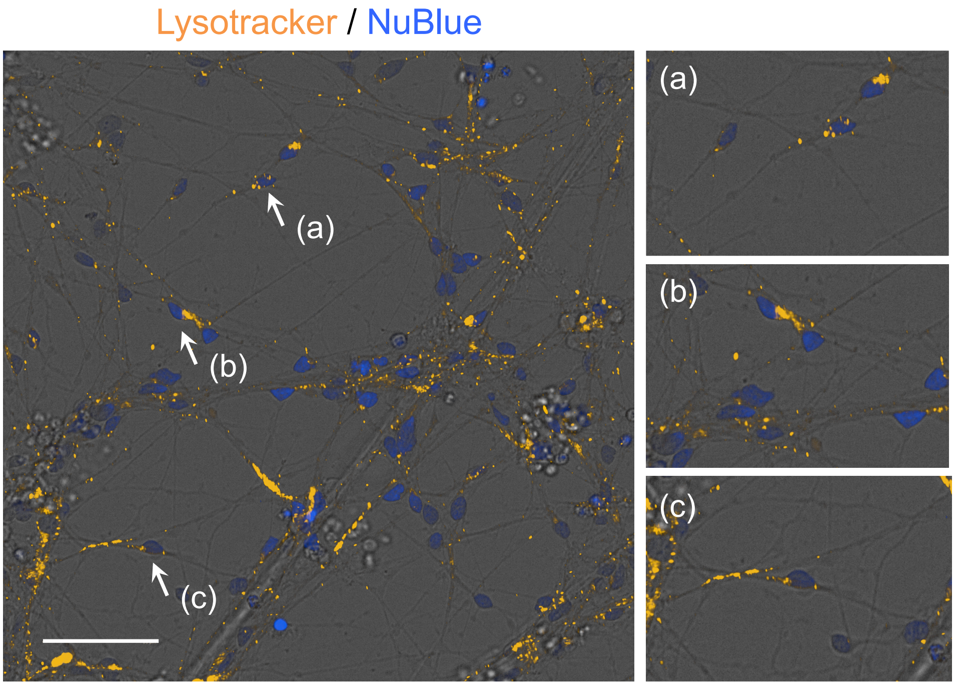

Figure S3. Lysotracker-positive vesicles were concentrated near somatodendritic region. Related to Figure 3. Representative images of iPSC-derived neurons stained for endogenous lysosomes with LysoTracker Red and nuclei labelled with NucBlue. Regions of interest (arrows) are magnified on the left panels (a, b and c). Scale bar, 50 µm.

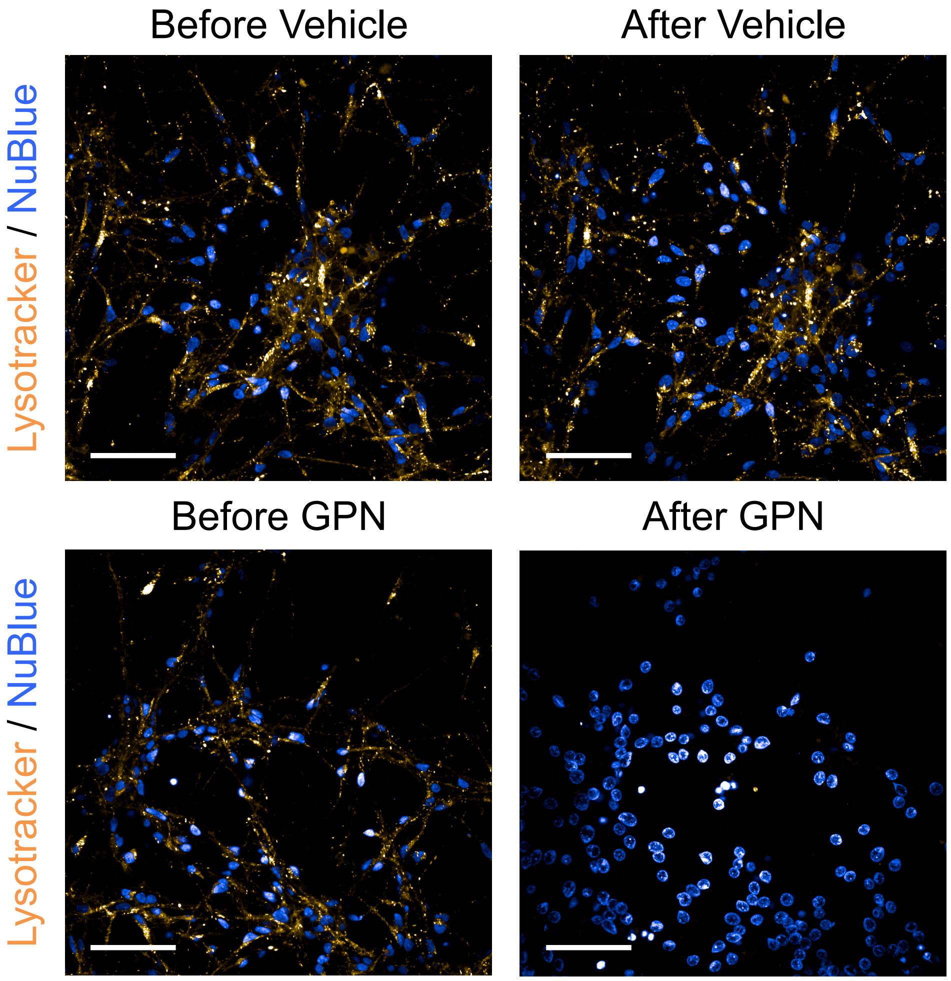

Figure S4. GPN abolishes LysoTracker staining by disrupting lysosomal membrane. Related to Figure 3. Representative images of iPSC-derived neurons stained for endogenous lysosomes with LysoTracker Red and nuclei labelled with NucBlue. Images were captured before and after adding either vehicle (DMSO) or 50 µM GPN. Scale bar, 50 µm.

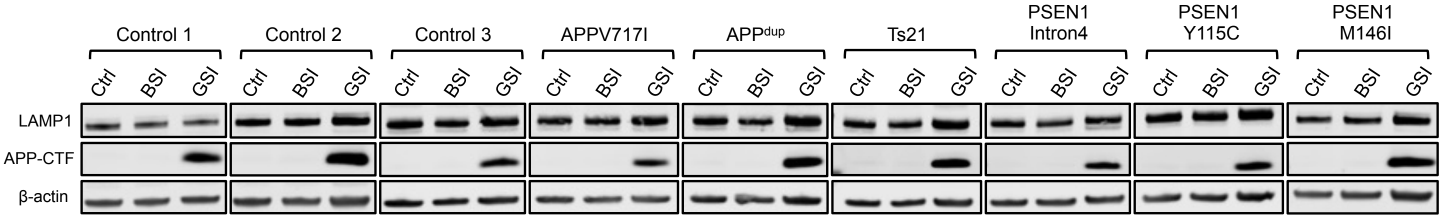

Figure S5. Manipulation of γ-secretase activity alters LAMP1 expression in genetic forms of AD. Related to Figure 6. Western blots performed on soluble protein extracts of neurons at day 90 post-neural induction. DAPT treatment (GSI) increases LAMP1 expression in all genotypes. By contrast, OM-99 (BSI) reduces total LAMP1 expression in the majority of genotypes.
